# Fluorescent Sensors for Biological Applications

**DOI:** 10.3390/s140917829

**Published:** 2014-09-25

**Authors:** Hui-wang Ai

**Affiliations:** Department of Chemistry, University of California Riverside, 501 Big Springs Road, Riverside, CA 92521, USA; E-Mail: huiwang.ai@ucr.edu

Fluorescence is one of the most important analytical methods used in biological studies. In the past decade or two, instrumentation in this field has greatly advanced, and now it is possible to detect single photons or fluorescent molecules [1,2], or break the Abbe diffraction limit to distinguish two points spaced less than 50 nm apart [3]. Concurrently, the development of improved fluorescent probes, which can be coupled with state-of-the-art instruments, has been equally important. This special issue on “fluorescent biosensors” in *Sensors* reports recent results from eight research groups in the field of sensor development. It includes three review articles, and six research articles reporting original results.

Guo and Tan review recent studies on the delayed fluorescence of plant photosystem II (PS II) [4]. When plants absorb light for photosynthesis, weak and long-lifetime fluorescence is observed from PS II. This dark fluorescence is a powerful indicator of plant photosynthesis rate, circadian, senescence and stress conditions. This reviewer article should be of great interest to the plant biology community, since a single measurement of dark fluorescence may provide “information on many aspects of plant physiology and environment” [4].

Ren and Ai summarize redox probes based on fluorescent proteins [5]. Since the 1990s, fluorescent protein-based technologies have been greatly expanded and revolutionized biology. In this paper, genetically encoded probes that can be utilized to detect redox biological processes are reviewed. Their properties, molecular mechanisms, advantages and pitfalls are discussed, providing rich information to biologists for the use of redox fluorescent probes in their studies.

Benčina provides a thorough overview of genetically encoded pH probes, also based on fluorescent proteins [6]. Both intensity-based and ratiometric pH probes are summarized. Their spectral properties, the pH of intracellular organelles, and instrument setups are also discussed. This review will assist researchers in choosing pH-sensitive fluorescent protein for their particular experiments.

In this special issue, Benčina and coworkers also contribute a research article [7], which reports a high-throughput method based on ratiometric flow cytometry for single-cell analysis of HIV protease activity. They first developed an HIV protease sensor from a pair of fluorescent proteins, mCerulean and mCitrine. The sensor was used for HIV protease inhibitor screening assays and intracellular quantitative detection of HIV protease activity. Ratiometric flow cytometry was also utilized to analyze large populations of cells for HIV protease activity. Finally, confocal microscopy was used to evaluate the sensor for spatiotemporal HIV protease activity in cells.

Bodart *et al.*, report the optimization of fluorescent protein-based ERK kinase probes, and utilized the new sensors showing improved dynamic ranges to image EGF stimulation of living cells [8]. Both the ratiometric mode and lifetime imaging were demonstrated with these new probes.

Campbell *et al.*, describe their mutagenesis studies on CH-GECO2.1, a red fluorescent protein-based Ca^2+^ sensor developed by the same group [9]. A large number of mutants were created and characterized, leading to the identification of a key residue, Gln163, involved in the conformational change that transduces Ca^2+^ binding into fluorescence changes. They also found that the interdomain linkers and interfaces are important for binding affinities of these chimeric Ca^2+^ sensors.

Aliaga *et al.*, report two synthetic coumarin-based “turn-off” fluorescent probes for the detection of Cu^2+^ and Fe^3+^ ions [10]. Both metal ions quenched the fluorescence of the two dye molecules. Epifluorescence microscopy was used to image Cu^2+^ and Fe^3+^ ions in human neuroblastoma SH-SY5Y cells.

Anderson *et al.*, describe a DNA hairpin-based system to report temperature and ionic changes [11]. They investigated base compositions, sequences, ionic strength, and other factors for their impact on hybridization kinetics and temperature sensitivity. These nanoscale sensors are expected to have broad applications.

Ostermann *et al.*, developed a signaling system based on two types of yeast cells, one to secrete the pheromone α-factor and the other to emit fluorescence upon activation by the pheromone α-actor [12]. They tested factors such as distance, response time, and ratio, for cells immobilized and separated in agarose hydrogel. From their experimental data, they derived a mathematical diffusion model for this system.

To end, I would like to thank all authors and reviewers for their contributions and hard work to put up together this special issue highlighting the current status of fluorescent biosensors. I strongly believe that, through scientific studies in various laboratories, the future of fluorescent biosensors will be even brighter and more exciting.
